# The Proper Motor Control Model Revealed by Wheelchair Curling Quantification of Elite Athletes

**DOI:** 10.3390/biology11020176

**Published:** 2022-01-23

**Authors:** Xiangdong Wang, Ruijiao Liu, Tian Zhang, Gongbing Shan

**Affiliations:** 1 School of Physical Education, Jimei University, Xiamen 361021, China; wangxiangdong@jmu.edu.cn; 2 Graduate School, Beijing Sport University, Beijing 100084, China; 2020210364@bsu.edu.cn (R.L.); 2019210228@bsu.edu.cn (T.Z.); 3 Biomechanics Lab, Faculty of Arts & Science, University of Lethbridge, Lethbridge, AB T1K 3M4, Canada

**Keywords:** biomechanics, generalizable control pattern, acceleration phase, stabilizing delivery phase, synchronized effort, sequential coordination

## Abstract

**Simple Summary:**

This is the first study to quantitatively explore the motor control of elite wheelchair curling athletes. It is known that, psychologically, wheelchair users are often not comfortable with their wheelchair motor skills and, therefore, hesitate to participate in sports/physical activities. For increasing exercise of this population, an effective learning/training program should firstly be developed. This study has chosen a suitable sport, i.e., wheelchair curling, and identified and described generalizable characteristics or “markers” among elite athletes. Such markers could provide effective ways of accurately identifying, evaluating, and communicating when learning the skill. Due to this being an under-investigated area, this study also shed new light on how to scientifically promote physical participation of wheelchair users.

**Abstract:**

Background: Wheelchair users are disadvantaged when it comes to accruing the benefits of physical activities. Hence, promoting various sports is crucial for keeping this population healthy. Since wheelchair curling can be played by individuals from a wide range of ages, strengths, and endurance levels, it has potential to improve wheelchair users’ well-being. Yet, hardly any motion studies exist. This study aimed to facilitate understanding of optimized control of wheelchair curling for promoting wheelchair users’ participation. Methods: Using motion capture technology, nine national-level athletes were tested. Kinematic parameters related to segment/joint control and their coordination were quantified for both slow and fast curling. Descriptive statistics (means and standard deviations) and correlation analysis were applied for characterizing the skill. Results: (1) Curling control consists of an acceleration phase and a stabilizing delivery phase; (2) the control of trunk, shoulder, and wrist are responsible for accelerating the rock; (3) elbow control is accountable for the accurate delivery of the rock; and (4) during the slow curling, a synchronized effort of trunk, shoulder, and wrist is used for accelerating the rock, while a sequential control among the segment/joints is applied in fast curling. Conclusions: The results supply valuable motor learning markers that could have a significant positive impact on the teaching and learning of wheelchair curling, as such, the findings have great potential for the health promotion of wheelchair users.

## 1. Introduction

According to the report of World Health Organization (WHO) on disability, over 65 million people live with a disability requiring the use of a wheelchair [1]. It is known that wheelchair use has a negative association with physical activity participation. As a consequence, wheelchair users are doubly disadvantaged for accruing the benefits of physical activity and exercise [2]. Studies have revealed that, due to the lack of physical exercise, wheelchairs users are at risk of obesity and cardiovascular problems [3,4,5,6,7]. Maintaining regular physical activity/exercise as appropriate is an effective prevention option [7,8,9,10]. Hence, promotion of various physical participations are crucial for keeping this population healthy.

Wheelchair users face a number of barriers to exercise. One of them is that they may not be comfortable with their wheelchair motor skills and, therefore, may not participate in wheelchair sports or physical activities [11]. Previous studies have revealed that physical activities are more important for people with disabilities relative to people without disabilities, and individuals who are physically active enjoy a range of benefits, spanning physiological, emotional, cognitive, and social categories [7,12]. Therefore, efforts to facilitate physical activity/exercise by wheelchair users should focus not only on interventions relating to personal assistance and assistive technology, but also on the selection of proper sport programs that foster participation in sports among wheelchair users for health promotion.

Introduced in 2000 at the World Handi Ski Championship in Crans Montana (Switzerland), wheelchair curling has increased markedly in popularity. Now, this sport is a Paralympic match practiced by athletes from dozens of countries [13]. This relatively new sport is unique for wheelchair users, because it can be played by a wide range of ages, strengths, and endurance levels, i.e., it can also be a new recreational physical activity for wheelchair users of varying motor ability levels, for different age groups. As such, this team sport has a great potential to improve wheelchair users’ health, fitness, and well-being by minimizing the existing negative influences among wheelchair users, such as low self-esteem, social isolation, and depression. A recent preliminary study has shown that this relatively new sport could offer a suitable alternative to sports already used in rehabilitation and in recreational activities for wheelchair users, because the subjects of the study subjectively reported improved physical feeling and an increase in motor control and strength [14]. Certainly, more studies are needed for drawing more unambiguous conclusions. 

From the exercise science point of view, one barrier for promoting the sport is related to the knowledge/information of the motor skill learning. A search in Web of Science (by applying the keywords: wheelchair, curling, and biomechanics) showed only one case study with a single subject dealing with the skill control/biomechanics of participating in wheelchair curling. The single-subject case study built a model of wheelchair curling [15] and performed thereafter a model-based optimization of curling skills [16]. It is known that the generalizability of a case study is always an issue. For a delimitation, a sample group of subjects should be cautiously selected in order to gain fundamental knowledge of wheelchair curling skills. Additionally, motor skills are often poor in the disabled. Thence, promotion programs on physical participation of wheelchair users should ensure that the motor skill learning does not become discouraged and cause a drop out of the learning before enjoying the physical benefit [17]. Previous studies have shown that science-based motor skill learning is an effective way for improving the efficiency of learning and/or training [18,19,20,21]. 

The dominant skill of wheelchair curling is control of the upper limbs [15,16,22]. A previous injury study has revealed that wheelchair curling remains a low-risk sport in terms of possible muscular-skeleton injury and the provision of injury prevention for chronic upper limb conditions seems appropriate [23]. One common causal factor leading to chronic upper limb injury is improper limb control during physical activities [24,25]. Given the current state of the knowledge on wheelchair curling, there is a clear need for investigating the proper upper limb control in order to develop efficient and effective motor learning strategies to promote the sport while minimizing or preventing injuries during skill learning/training. Understanding proper limb control in various sports requires the development of a complete and quantitative biomechanical/kinematic perspective of the chosen sport [26,27,28,29]. Accurate motion analysis is foundational to developing this perspective and to the design of practices that focus on economic training to reduce the accumulation of small micro-traumas that cause muscular-skeleton injuries [30]. Further, an examination of a group of professional wheelchair curling athletes may lead to generalizations and/or optimization for the motor skill learning. As such, this paper initiated a quantitative kinematic analysis of nine national-level wheelchair curling athletes.

The most common technique of wheelchair curling is that the trunk supplies a stable base for accurate control of the curling arm and hand, i.e., a dynamic chain control/coordination related to trunk, upper arm, lower arm, hand, and curling delivery stick [22]. Quantitative kinematic analysis of the movement control provides a basis for comprehension of the biomechanical demands of the skill control. Consequently, this study focused on revealing kinematic characteristics of the dynamic chain control. Specifically, the study examined: the initial posture; the ROM for the trunk, shoulder, elbows, wrists; the timely coordination among the segment/joints; and the dominant factors influencing the release speed of the rock. The aim was to facilitate a better understanding of the optimized control characteristics for wheelchair curling in a manner that will be directly communicated with practitioners. Such information should help practitioners improve the learning efficiency and effectiveness in their training programs and better develop training plans for wheelchair users. We speculated that faster and more effective skill acquisition will increase enthusiasm for, and participation rates in, the wheelchair sport. As such, the results can be applied in health promotion, allowing practitioners to quantify and improve the disparate parameters characterizing movement in musculoskeletal system of wheelchair users in their daily lives.

## 2. Materials and Methods

In wheelchair curling, the chair is static. As such, wheelchair curling biomechanics should involve understanding the coordination between upper body and the delivery arm during delivery of the stone. To increase the effectiveness of motor skill learning/training, it is vital to obtain proper (i.e., stable/optimized) kinematic characteristics of the skill. This study employed a repeated-measures design (classified as quasi-experimental), i.e., no effect of an intervention on a selected outcome or comparison of outcomes between experimental and control groups. The design is widely used in identifying the well-trained/stable motor control patterns that exist in national-/international-level elite athletes to obtain control parameters that can be used for improving motor learning/training in coaching practice [31,32,33,34,35]. Therefore, the following design elements were selected for the pilot study. 

### 2.1. Subjects

The research design of this study required truly elite subjects who had experience performing at the world championship and Olympic level. Due to the limited number of these athletes and the practical difficulty for the recruitment of these elite subjects, the subject number of this type study is commonly small, ranging from 2 to 9 [31,32,33,34,35]. In this pilot study, nine Paralympic athletes (eight males and one female) from the Chinese national team were successfully recruited.

It is a research policy of the China Institute of Sport Science that an ethics committee approval is not required for video-based motion analyses (i.e., non-interventional, observation-type studies) during training sessions of national team elites. With the support of the Chinese national wheelchair curling team, our research team had the opportunity to collect video data in the training gym of the national team during their training sessions. Such a real-life data collection setting can eliminate constraints induced by laboratory-based investigations on athletes’ performance. As such, the naturalistic control pattern (i.e., the trained, optimized control pattern) can be revealed for defining the proper motor control for learners. 

### 2.2. Protocol 

The data collection was performed on a standard curling sheet. Two common stone delivery techniques (i.e., slow and fast) were tested. Each subject performed three throws per technique, which resulted in 54 trials in total. 

**Figure 1:**
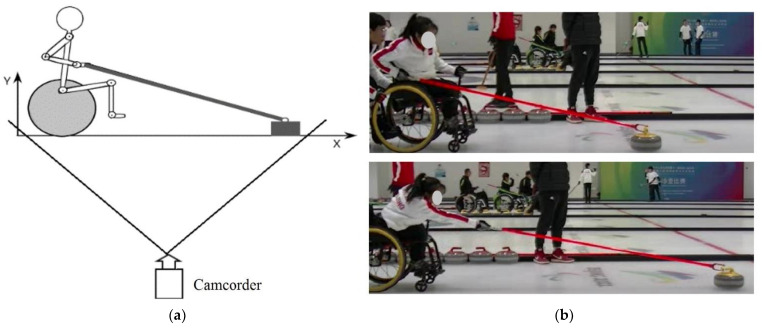
Kinematic data collection: (**a**) the set-up for data collection; (**b**) the sample frames showing the beginning of the delivery and the release of the stone.

In wheelchair curling, an athlete must stabilize the non-delivery arm by holding onto the post or the wheel on the non-delivery side and the delivery control happens mainly in the sagittal plane, therefore 2D motion analysis can be applied for kinematic quantification. A JVC camcorder (GZ-R465BAC, Yokohama, Japan) was used for the motion capture at a capture rate of 50 frame/s. The camcorder was placed on the delivery side, 5 m from the subject. The height of the main optical axis of the camcorder was set roughly at the same height of the subject’s elbow (Figure 1). KEXING Motion Analysis Software (KEXING Ltd., Beijing, China) was applied to obtain kinematic data. Using the manufacturer’s specified guidelines, calibration resolution yielded results accurate < 2 mm. The second-order bi-directional low-pass digital filter (6 Hz) was applied to smooth the raw coordinate data. 

Three stone-related parameters, i.e., rock release speed, rock throwing time, and distance covered by rock during throwing, were chosen to measure the delivery/throwing quality. In order to reveal the control characteristics of the coordination between the upper body and the delivery arm during throwing, four kinematic parameters for trunk, shoulder, elbow, and wrist were selected for a quantitative analysis. The four kinematic parameters were initial angle, range of motion (ROM), maximum angular velocity (Max V_angular_), and time at Max V_angular_. 

### 2.3. Data Analysis 

For data analysis, the means and standard deviations (SD) were calculated for both throwing quality and control/coordinate parameters. The descriptive statistics (mean ± SD) were used to illustrate the characteristics of the selected parameters. Pearson correlation analyses between rock release speed and the coordinate variables were performed to determine the parameters highly influencing throwing quality. A commonly used r-value interpretation in sports biomechanics and human kinetics [36] was applied for the r-value cut-off to show an accepted relationship between the segmental/joint control and the rock release speed, i.e., a moderate relationship or higher (|r| ≥ 0.5). Given that there are four segments involved in the skill control, multiple corrections would possibly result in a type I error. Hence, the Bonferroni adjustment was applied to change the significance level from *p* = 0.05 to *p* = 0.0125 for determining the possible influences of segmental/joint control on the release speed of the rock.

Due to the small sample size, the Shapiro–Wilk Test was first performed to test the normality of the collected data; if the data were normal, the paired T-test between the slow and fast throwing was conducted to detect changes in control patterns induced by the change of curling techniques. In addition, the power analyses on the T-test results were performed to make sure that the probability of the detect effects were higher. All the statistical analyses were done by using IBM SPSS Statistics 23 (IBM Japan, Tokyo, Japan) and the significance level was set at *p* = 0.05. 

## 3. Results

The Shapiro–Wilk Test confirmed the normality of the collected data (*p* > 0.10) and the power analyses showed higher probability (0.83–0.99) of the detected effects. Table 1 shows the throw-quality characteristics of the slow and the fast curling. On average, slow and the fast curling had rock release speeds of 2.10 m/s and 2.93 m/s, rock throwing times of 0.68 s and 0.60 s, and distance covered by the rock during throwing of 0.81 m and 1.01 m, respectively. T-tests revealed highly significant differences (*p* < 0.01) between the two throwing techniques. In comparison to slow curling, fast curling increased the release speed by about 40%, shortened the throwing time by about 13%, and enlarged the distance during throwing by about 25%.

Generally, the curling techniques had no significant influence on coordination, i.e., on the initial angle and the ROM of trunk, as well as on the ROM of the shoulder, elbow, and wrist on the curling side (*p* > 0.05). The following kinematic characteristics are revealed in Table 2: body control was initiated by the trunk around 70° in the horizontal direction (the slow: ~68°, the fast: ~70°), the shoulder extension (negative value) around 35° (the slow: 35°, the fast: 38°), the elbow angle around 65° (the slow: ~65°, the fast: ~69°), and the wrist abduction angle around 175° (the slow: ~175°, the fast: ~171°). The ROM was around 25° (the slow: ~21°, the fast: ~27°) for the trunk control, around 145° (the slow: ~140°, the fast: ~148°) for the shoulder control, around 85° (the slow: ~85°, the fast: ~81°) for the elbow control, and around 10° (the slow: ~12°, the fast: ~9°) for the wrist control. The wrist control was relatively small, with large variations among individuals. T-tests showed that significant differences existed in speed–time control between the two curling techniques. On average, the max angular velocities of trunk, shoulder, and elbow during fast throwing were 51%, 18%, and 27% faster than those during slow throwing, respectively. Regarding the timely control, the max angular velocity of the trunk during fast throwing occured 53% earlier than that of slow throwing, while a reverse control (37% delay) is found in the wrist control. 

The correlation analysis revealed a moderate positive relationship between max rotatory velocity of trunk, shoulder, and elbow and the rock release speed (Table 3). A strong negative relationship between the timely control of the trunk and the rock release speed was observed. The results indicate that the timely coordination between trunk, shoulder, and elbow influences the rock release speed obviously. 

## 4. Discussion

The overarching objective of this study aimed to foster participation in sports among wheelchair users. Wheelchair curling, a relatively new sport, has been chosen because it has great potential to offer a suitable alternative that can be used in rehabilitation and in recreational activities for wheelchair users [14]. To this end, the current study aimed to accomplish two specific objectives related to motor learning of the sport: (1) to describe the kinematic characteristics of the trunk and curling arm, taking into account the influence of curling techniques (i.e., slow or fast), and (2) to reveal the dominant control factors (where possible) that could be used to develop a science-based motor skill learning for improving the efficiency of learning and/or training. 

**Figure 2:**
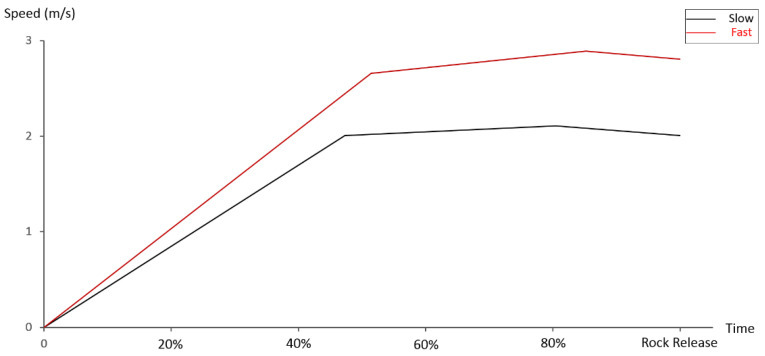
The development of the average rock speed over the course of curling.

Overall, the initial posture, the ROMs of trunk and joints involved, and their coordination come into play in some critical areas, and as such balance, integration, and sequencing of the complex set of movements are required for a quality delivery of the rock. This study has revealed the existence of general control patterns related to the initial postures and the ROMs of the trunk/joints of the curling arm regardless of curling techniques. The control pattern can be summarized as: (1) the curling control consists of an acceleration phase and a stabilizing delivery phase; (2) the control of trunk, shoulder, and wrist are responsible for accelerating the rock; and (3) control of the elbow is accountable for the accurate delivery of the stone. In combination with Figure 2, the general control pattern can be drawn from the data of Table 2. These results clearly indicate, for both techniques, that there is a fast increase of the rock speed in the first half of the curling and a relatively stable rock speed in the second half of the curling; further, the max angular velocities of trunk, shoulder, and wrist appeared in/close to the first half, while the maximum angular velocity of elbow emerged toward the end of the curling. The finding of the elbow control indicates that the lengthening of the elbow control time would produce the most accurate kinematic trajectories. It is well known that speed and accuracy control are two fundamentals of wheelchair curling; therefore, this finding could enable practitioners to postulate the skill control in training beginners, and could be also considered an important measure in evaluating teaching/training effect. 

**Figure 3:**
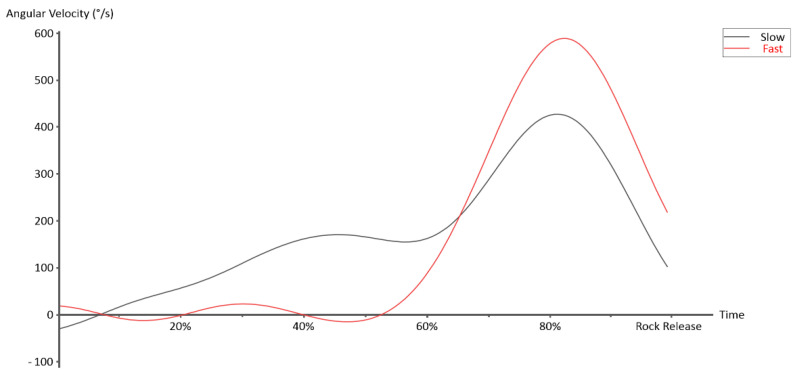
Typical elbow controls over time of the two curling techniques.

Concentrating on the influences of the curling techniques, we observed a significant impact of curling speed on timely coordination among the trunk, shoulder, elbow, and wrist. One notable marker was identified: a synchronized effort of trunk, shoulder, and wrist is used for accelerating the stone in the first half during the slow curling, while a sequential control among the segment and the joints is clearly apparent in fast curling. The data in Table 2 indicate that the rotatory velocities of trunk, shoulder, and wrist reach the maxima almost at the same time during the slow curling, yet such a control disappears in the fast curling. Further details obtained from the typical elbow control over time even confirmed that, during slow curling, the elbow extension jointly contributes to the acceleration of the stone, i.e., the first half of the curling, but not during fast curling (Figure 3). Therefore, all segments/joints are dedicated to the rock acceleration in slow curling and the further rotatory control of the elbow is responsible for the accuracy of the delivery. In contrast to the slow curling, a sequential rotary control from the trunk and shoulder to the wrist accelerate the rock in the first phase and the last extension of the elbow in the second phase is in control of the accuracy of the delivery. Previous studies of various motor skills have shown that establishing the sequential motor control is challenging [19,20,21,26]. It requires a clear instruction and long-term training. The findings of the timely unique controls induced by the curling techniques provide valuable markers for the teacher and learner to evaluate their training effects and remediate, if necessary. 

Both selecting accessible sports and maintaining participation in the sports play vital roles in health promotion among wheelchair users. One approach to promotion is to increase the motor learning efficiency and effectiveness. Hence, for reaching effective learning, characterizing the wheelchair curling should involve objective scientific analysis of requisite fundamentals to identify, describe, and generalize desired movement control. Using movement analysis technology to capture and analyze the motion, the current study has established wheelchair curling characteristics of high-level athletes. The obtained general control patterns can then be used to direct attention to specific controls that learners (novices) need to assimilate into their technique. These scientific fundamentals can be understood in such a manner that skill-transfer can occur, regardless of individual differences. One should keep in mind that an inappropriate introduction/self-learning (i.e., trial-and-error learning) of training modalities before a learner has developed the required body segments’ controls represents a non-productive investment of training time and distracts from the development of proper motor skills that are both essential and appropriate at the three stages (i.e., cognitive, associative, and autonomous) of the motor learning process [37]. The probable consequences are predictable—full potential, as such interest and motivation for practice, may never be attained and, in a negative scenario, early withdrawing from regular participation may result. Hence, from both health and the motor learning point of view, the current study would contribute to the health promotion and, possibly, the long-term wellness of wheelchair users. 

Practically, training/motor learning for beginners could begin with slow curling, aiming to establish and stabilize the control pattern with the rock release speed around 2 m/s. Biomechanically, real-time feedback (the rock release speed) training could accelerate learning and feedback can now be easily obtained by attaching an inertial measurement unit (IMU, a wireless small sensor) onto the rock [38]. After stabilizing the slow curling technique, the learning of fast curling can be started with the release speed around 3 m/s. Motor learning from less complex to complex motor control will simplify the learning process [37]. As such, the stepwise feedback training would make it easier to establish the conceptual recognition of control differences induced by slow and fast curling, and therefore, to improve the learning efficiency.

This is the first study that has quantitatively explored the kinematics of a group of elite wheelchair athletes, providing an objective external view of the curling process and factoring the motor skill into the results. It is understandable that there are limitations associated with this study. There are two obvious ones. Firstly, it is known that long-term training leads to highly stable control patterns in individual athletes of professional caliber [30]. Although the results obtained from elite athletes are highly reliable, the development process of the stable skill is missing. In other words, the current study has identified the proper motor control characteristics, but not the motor learning process. From the motor learning perspective, it gives rise to speculation that comparative studies between professional-level and less advanced athletes might lead to improvements in pedagogical methodology by identifying key factors accelerating the repeatability of the proper control. As implied by the famous quote from the legendary football coach Vince Lombardi, “Practice doesn’t make perfect. Perfect practice makes perfect” [39]. In order to develop more detailed descriptors in learning/training practice, comparative investigations should be launched in future studies. Secondly, due to the body structure differences induced by gender [40,41], there might be gender-based control pattern variation. In order to apply proper learning and training for female wheelchair users, a quantitation of elite female athletes as well as comparisons between males and females must be conducted in the future studies. 

## 5. Conclusions

The current study has established that some generalizable characteristics exist among highly trained elite athletes. Using these, several markers have been identified and quantitatively described: postural, positional, and time-based dynamic coordination among segment/joints. The study has unveiled the following results: (1) curling control consists of an acceleration phase and a stabilizing delivery phase; (2) the control of trunk, shoulder, and wrist are responsible for accelerating the rock; (3) elbow control is accountable for the accurate delivery of the rock; and (4) during slow curling, a synchronized effort of trunk, shoulder, and wrist is used for accelerating the rock, while a sequential control among the segment/joints is applied in fast curling. Such markers could have a significant positive impact on the teaching and learning of wheelchair curling, regardless of physical and/or age differences. In short, the findings have great potential for the health promotion of wheelchair users.

## Figures and Tables

**Table 1:** The comparison of throwing quality between the slow and the fast curling.

Rock Control	Slow	Fast	Difference ^1^
Rock release speed (m/s)	2.10 ± 0.11	2.93 ± 0.17	39.25% **
Rock throwing time (s)	0.68 ± 0.07	0.60 ± 0.08	−12.70% **
Distance covered by rock during throwing (m)	0.81 ± 0.15	1.01 ± 0.12	24.83% **

^1^ The % difference = (the value of fast – the value of slow)/the value of slow; **—*p* < 0.01.

**Table 2:** The comparison of throwing control characteristics between the slow and the fast curling.

Condition	Control Parameter	Trunk	Shoulder	Elbow	Wrist
Slow	Initial angle (°)	67.78 ± 7.00	−35.45 ± 25.50	64.61 ± 11.50	175.23 ± 11.61
	ROM (°)	21.31 ± 12.79	140.15 ± 26.33	84.94 ± 12.82	12.43 ± 7.91
	Max V_angular_ (°/s)	59.29 ± 24.82	3553.54 ± 616.50	351.52 ± 41.43	132.03 ± 43.07
	Time at max V_angular_ (%)	48.06 ± 17.67	49.89 ± 22.62	80.43 ± 8.11	47.31 ± 24.69
Fast	Initial angle (°)	70.40 ± 5.82	−38.25 ± 19.95	68.83 ± 5.02	171.16 ± 11.81
	ROM (°)	27.44 ± 9.28	147.63 ± 18.80	81.06 ± 10.17	8.79 ± 17.54
	Max V_angular_ (°/s)	89.72 ± 23.04	4196.27 ± 385.92	446.16 ± 74.09	153.39 ± 72.83
	Time at max V_angular_ (%)	22.53 ± 18.83	51.41 ± 15.80	85.18 ± 10.49	64.70 ± 21.15
Difference	Initial angle	-	-	-	-
	ROM	-	-	-	-
	Max V_angular_	51.33% **	18.09% **	26.92% **	-
	Time at max V_angular_	−53.11% **	-	-	36.76% *

*—*p* < 0.05, **—*p* < 0.01.

**Table 3:** The influence of segmental/joint control on the release speed of the rock revealed by correlation analysis (r values, *p* < 0.0125).

Control Parameter	Trunk	Shoulder	Elbow	Wrist
Initial angle (°)	-	-	-	-
ROM (°)	-	-	-	-
Max V_angular_ (°/s)	0.51	0.55	0.50	-
Time at max V_angular_ (%)	−0.67	-	-	-

## Data Availability

The data presented in this study are available on request and after appropriate IRB approvals.
